# A Proposal for Source Tracking of Fecal Pollution in Recreational Waters by Pulsed-Field Gel Electrophoresis

**DOI:** 10.1264/jsme2.ME13075

**Published:** 2013-11-19

**Authors:** Takashi Furukawa, Yoshihiro Suzuki

**Affiliations:** 1 Department of Civil and Environmental Engineering, Oita National College of Technology, 1666, Maki, Oita, 870–0152, Japan; 2 Department of Civil and Environmental Engineering, Faculty of Engineering, University of Miyazaki, 1–1, Gakuen Kibanadai-Nishi, Miyazaki 889–2192, Japan

**Keywords:** pulsed-field gel electrophoresis (PFGE), fecal pollution, enterococci, coastal recreation area, microbial source tracking

## Abstract

This study aimed to identify specific river sources of fecal contamination by applying pulsed-field gel electrophoresis (PFGE) to environmental water samples from a recreational beach in Japan. The genotypes of all *Enterococcus faecium* and *Enterococcus faecalis* strains used as indicators of fecal pollution on the recreational beach and rivers were analyzed by PFGE, and the PFGE profiles of the strains were classified at a 0.9 similarity level using dendrogram analysis. PFGE types of *E. faecium* isolated from Sakai River or urban drainage were classified in the same cluster. Therefore, the probable sources of fecal pollution on the recreational beach were Sakai River and urban drainage. The approaches for microbial source tracking employed in this study used PFGE with *Enterococcus* species as an indicator can be a potential tool to specify the source(s) of fecal pollution and contribute to improved public health in coastal environments.

Fecal contamination of water environments by water-borne pathogens such as *Escherichia coli* O157:H7, *Cryptosporidium*, *Giardia*, and noroviruses is a serious problem to public health (22, 28). Concern about human infections and the detection of these pathogens in aquatic environments has been increasing (25). Coastal areas often receive many different types of water that can be affected by domestic and industrial human activities. Consequently, fecal microorganisms that flow in rivers to coastal areas have a wide variety of origins that are classified as point sources or nonpoint sources (24). On the other hand, coastal areas play an important role in human recreation and shellfish production, and the contamination of coastal surface waters could be a serious threat to human health. Therefore, identifying the source of fecal pollution is important to contribute to the improvement of bacterial water quality and ensure public health in water environments.

Potential indicators of fecal pollution include fecal coliforms, *E. coli*, enterococci, and other fecal micro-organisms (*Bacteroides* spp., *Bifidobacterium* spp., and coliphages). Among these species, fecal coliforms, *E. coli*, and enterococci have been historically used as indicators of choice when monitoring coastal water quality (31). However, in Japan, there are no standards concerning bacterial water quality in aquatic environments. It has been reported that high densities of *E. coli* and enterococci in recreational surface waters are more strongly correlated with swimming-associated gastrointestinal disease than high densities of fecal coliform (4). The genus *Enterococcus* has been recommended as an indicator of fecal pollution because of its long-term survival and low growth rate in aquatic environments (14, 16).

Many studies have attempted to develop microbial source tracking (MST) methods using different indicators of fecal pollution (8, 17, 21, 23). Various phenotypic and genotypic methods classified as library-dependent and library-independent methods have been examined for MST. Numerous studies provide useful knowledge and information related to MST methods, including review articles (3, 22, 26, 28), guidance documents (34), and comparative research on genotypic and phenotypic methods (1, 9, 29). However, there is still no standard method for source tracking of fecal pollution.

Pulsed-field gel electrophoresis (PFGE) is a DNA fingerprinting method that can distinguish different strains within a species by comparing genotypic characteristics. It is an analytical technique in the field of molecular epidemiology (2, 27), where this method has been traditionally used for identifying the route and source of infection in nosocomial infections and for specifying bacteria that are responsible for food illness (7, 19). PFGE has also been applied to source tracking of *Salmonella* contamination in marine environments (18). PFGE has extremely high sensitivity, reproducibility, and discrimination ability compared with other MST methods (22).

We have previously proposed MST using PFGE and have strongly indicated its ready availability (5). In a case study conducted on Aoshima Beach, Japan, the fecal pollution source for a swimming beach was identified among five rivers by MST using PFGE with *Enterococcus faecium* as an indicator of fecal pollution (6). However, it is necessary to demonstrate its adaptability and geographical stability in coastal areas to establish PFGE as an MST method. *Enterococcus faecalis* is also important as an indicator of fecal pollution along with *E. faecium*. In certain cases, *E. faecalis* is the dominant *Enterococcus* species in river and coastal waters. Therefore, in this study, MST was performed with two species of major enterococci, *E. faecium* and *E. faecalis*, collected from a recreational beach and entirely different from those collected from Aoshima Beach and rivers near the beach. The source of fecal pollution was tracked by comparing dendrograms of PFGE types obtained from the two *Enterrococcus* species.

## Materials and Methods

### Outline of source tracking

**Fig. 1:**
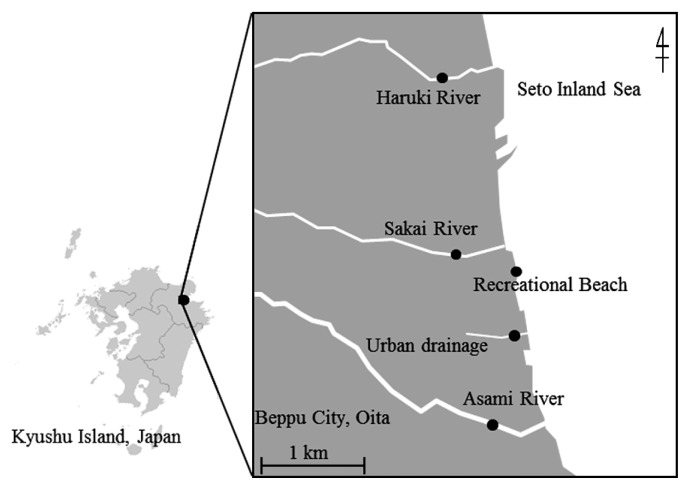
Sampling stations.

This demonstration of fecal pollution source tracking using PFGE was conducted at a recreational beach in Oita, Japan, facing the Seto Inland Sea. The beach (latitude: 33.27°N; longitude: 131.50°E) and rivers are shown in Fig. 1. There is no official bathing season at the beach; however, it is an important recreation area because several events (festivals, fireworks) are held here during the summer. The area covered and lengths of the three rivers, *i.e.*, Haruki, Sakai, and Asami rivers, were 8.6 km^2^ and 6.27 km, 12.2 km^2^ and 6.90 km, and 10.9 km^2^ and 5.03 km, respectively. The distance of the Haruki River, Sakai River, Asami River, and urban drainage from the recreational beach is approximately 2.5, 0.6, 1.3, and 0.5 km, respectively.

Sampling was conducted on July 21, 2009. The coastal water sample was collected from surface water at a depth of approximately 50 cm near the water’s edge on the recreational beach. River water samples were collected from the surface of each river. The sampling points of river water were within approximately 500 m of the estuary for each river. There was a slight effect of seawater mixing at the river sampling points. A water sample was also collected from urban drainage, a candidate fecal pollution source, passing through a residential area near the beach. In all, 5 surface water samples were stored in sterile 1-L polyethylene bottles and immediately transported to the laboratory, where they were tested for fecal bacteria and water quality.

### Water quality parameters

We analyzed enterococci, coliforms, and *E. coli* in each water sample. pH, electrical conductivity (EC), and turbidity were also determined as major water parameters using a pH meter (HM-30G; TOA DKK, Tokyo, Japan), conductivity meter (CM30S; TOA DKK), and turbidity meter (SEP-PT-706D; Mitsubishi Kagaku, Tokyo, Japan), respectively. Salinity was calculated from EC (20), and water temperature was determined using a stick thermometer during sampling.

### Bacterial counts

*Enterococcus* counts were determined by the membrane filtration (MF) method (32). One hundred milliliters of each water sample were filtered in triplicate using a 0.45-μm pore membrane filter (47 mm diameter, sterile, mixed cellulose ester; Advantec, Dublin, CA, USA) and incubated on membrane-Enterococcus indoxyl-β-d-glucoside (mEI) agar plates for 24 h at 41±1.0°C. After incubation, blue halo colonies on the filter were determined as enterococci. Coliforms and *E. coli* were analyzed using the Colilert-18 Test Kit (Idexx Laboratories, Westbrook, ME, USA) according to the manufacturer’s instructions. The detection limits of enterococci, coliforms, and *E. coli* were 1.0 CFU 100 mL^−1^, 1.0 MPN 100 mL^−1^, and 1.0 MPN 100 mL^−1^, respectively.

### Isolation of enterococci

After the *Enterococcus* count, a single blue halo colony from each mEI agar plate was randomly streaked on a Todd Hewitt agar plate (TH agar plate; Becton Dickinson, Franklin Lakes, NJ, USA; added agar 1.5%), followed by incubation for 24 h at 37 ± 1.0°C. One hundred and fifty colonies of *Enterococcus* strains from the beach and 100 colonies from each of the rivers (Haruki, Sakai, and Asami rivers) and urban drainage were isolated. Identification tests were performed on all *Enterococcus* strains isolated from each water sample.

### Identification of *Enterococcus* strains

*E. faecium* and *E. faecalis* were identified using PCR and Api 20 Strep (BioMerieux, Lyon, France). All the isolated *Enterococcus* strains were identified by PCR, and the strains identified as *E. faecium* or *E. faecalis* by PCR were subsequently confirmed using Api 20 Strep.

Genomic DNA was extracted from a single colony on the TH agar plate using the Insta Gene Matrix (Bio-Rad, Hercules, CA, USA) according to the manufacturer’s instructions. The extracted DNA was stored at −20°C until further use. 16S–rRNA-based PCR identification was performed using Takara Ex Taq (Takara, Otsu, Japan). The 20-μL PCR mixture contained 2.0 μL of 10×Ex Taq Buffer, 1.6 μL dNTP mixture (2.5 mmol L^−1^ each), 2.0 μL forward and reverse primers (10 μmol L^−1^), 0.2 μL Ex Taq (5 U μL^−1^), 4.2 μL sterile distilled water, and 8.0 μL template DNA extracted from an *Enterococcus* strain. The PCR amplification programs used were as follows: initial denaturation at 95°C for 4 min, amplification of products using 30 cycles of denaturation at 95°C for 30 s, annealing at 54°C (*E. faecium*) or 62°C (*E. faecalis*) for 1 min, and elongation at 72°C for 1 min. Amplification was followed by a final extension at 72°C for 5 min. PCR products were separated by electrophoresis on a 1.5% 1×Tris/borate/EDTA (TBE) agarose gel. The amplified DNA bands were visualized after ethidium bromide staining using UV light. The estimated band lengths were 215 base pairs (bp) with primers Efm-F (5′-gaaaaaacaatagaagaattat-3′) and Efm-R (5′-tgcttttttgaattcttcttta-3′) used for identification of *E. faecium* (11), and 518 bp with primers Efs-F (5′-gccactatttctcg gacagc-3′) and Efs-R (5′-gtcgtccctttggcaaataa-3′) used for identification of *E. faecalis* (13).

Subsequently, the *Enterococcus* strains identified as *E. faecium* or *E. faecalis* by PCR were analyzed using Api 20 Strep according to the manufacturer’s instructions. Using both PCR and Api 20 Strep, *Enterococcus* strains were identified as *E. faecium* or *E. faecalis*, and the genotypic characteristics of these strains were analyzed by PFGE.

### PFGE

To elucidate the genetic relationship among the *Enterococcus* strains, PFGE was performed using the CHEF Bacterial Genomic DNA Plug Kit (Bio-Rad) according to the manufacturer’s protocol, with a slight modification. In brief, 8–10 colonies from the TH agar plates incubated for 24 h at 37°C were suspended in 1.0 mL sterilized physiological saline in a 1.5-mL microcentrifuge tube. The tube was centrifuged for 3 min at 13,523×*g* (Centrifuge 5424; Eppendorf, Hamburg, Germany), and the supernatant was discarded. The pellet was resuspended in 150 μL cell suspension buffer. The suspension was mixed with 3.0 μL lysozyme (25 mg mL^−1^), 3.0 μL lysostaphin (2.0 mg mL^−1^; Wako Pure Chemical, Osaka, Japan), and 3.0 μL mutanolysin (≥4,000 U mg^−1^; Sigma-Aldrich, St. Louis, MO, USA), followed by incubation for 10 min at 37°C. Next, the suspension was mixed with 150 μL of liquid 2% CleanCut agarose before it was poured into plug molds. The sample plug was then incubated for 4 h at 37°C in 500 μL lysozyme buffer containing 10 μL lysozyme, 10 μL lysostaphin, and 10 μL mutanolysin. The plug was treated with proteinase K (>600 U mL^−1^) for 24 h at 50°C, after which the plugs were washed 5 times using 1 mL of 1×wash buffer for 30 min with rotation in a microtube rotator (MTR-103; As One, Osaka, Japan). The DNA embedded in each plug was digested with the restriction enzyme *Sma*I (25 U plug^−1^) in 300 μL *Sma*I buffer for 20 h at 25°C, after treatment with 1 mL of 10-fold–diluted (0.1×) wash buffer followed by 500 μL *Sma*I buffer.

DNA fragments were separated for 20 h at 14°C on 1% pulsed-field certified agarose gel (Bio-Rad) in 0.5×TBE buffer, with a switch ramp time from 5.3 to 34.9 s at a 120° angle, using a CHEF DR II system (Bio-Rad). The sizing ladder used for PFGE was a lambda DNA ladder with a range of 48.5 kb–1.0 Mb (Takara).

### Dendrogram analysis of PFGE patterns

Dendrogram analysis of band-based PFGE patterns was performed using a Gene Profiler (Scanalytics, Buckinghamshire, UK). Levels of similarity between fingerprints were expressed as Dice coefficients, which were calculated by determining the ratio of twice the number of bands shared by two patterns to the total number of bands in both patterns. PFGE patterns were clustered by the unweighted pair group method with arithmetic mean (UPGMA). In the dendrogram analysis of PFGE fingerprints, some strains that belong to the same cluster with a 0.9 similarity level should be considered as related species (30).

## Results and Discussion

### Major water parameters and bacterial counts

The major water parameters (pH, water temperature, salinity, and turbidity) and bacterial counts (enterococci, coliforms, and *E. coli*) are shown in Table 1. pH (7.3–7.7) and water temperature (28.1–28.7°C) were not considerably different at each sampling station. However, turbidity varied considerably among sampling stations. The turbidity of the beach sample was low compared with that of the river water samples. High turbidity was detected in Sakai (12.2 degrees) and Asami (18.4 degrees) rivers. The salinity at the beach was 28.4 psu, and salinities of the river water samples ranged from 0.1 to 0.2 psu, indicating that the sampling points on each river were not influenced by the inflow of seawater.

A high *Enterococcus* count was detected in the water sample from the beach (2.6×10^2^±10.8 CFU 100 mL^−1^, mean±S.D., *n*=3). The counts of coliforms (63 MPN 100 mL^−1^) and *E. coli* (below detection limit) detected in the water sample from the beach were relatively low compared with that of the enterococci. The *Enterococcus* counts were high in the samples from Haruki (5.2×10^2^±48.1 CFU 100 mL^−1^) and Asami (3.8×10^2^±75.1 CFU 100 mL^−1^) rivers. The *Enterococcus* counts of the samples from Sakai River and urban drainage were low compared with those of the other three samples. The coliform counts of water samples from all rivers were extremely high, *i.e.*, >10^3^ MPN 100 mL^−1^. The *E. coli* counts of all river water samples were <100 MPN 100 mL^−1^. *Enterococcus* counts were lower than the coliform counts, with the exception of those of samples from the beach.

On the beach, enterococci were detected at a concentration that exceeded the present single sample limit for enterococci (104 CFU 100 mL^−1^) recommended by the US Environmental Protection Agency for marine recreational water (31). However, a low concentration of coliforms and no *E. coli* were detected in the water sample from the beach. The *Enterococcus* counts of all river water samples exceeded the single sample limit for fresh water (61 CFU 100 mL^−1^) (33). High counts of enterococci were detected in Haruki and Asami rivers. Extremely high coliform counts were also detected in all river water samples. Overall, the sampling stations where the counts of fecal bacteria in the water samples were comparatively high may be polluted by pathogenic microorganisms derived from human and/or animal feces. However, simply monitoring bacterial water quality cannot provide detailed information about the source(s) of fecal pollution that can degrade water quality in coastal recreation areas.

### Identification of fecal indicator bacteria

Identification of *E. faecium* and *E. faecalis* was performed with PCR and Api 20 Strep, using the 150 *Enterococcus* strains isolated from the water sample of the beach (Table 2). Among these 150 strains from the beach, 94 and 22 strains were identified as *E. faecium* and *E. faecalis*, respectively, by PCR. Then, these 94 *E. faecium* and 22 *E. faecalis* strains were confirmed using Api 20 Strep. Eighty-nine of the 94 *E. faecium* strains were also identified as *E. faecium* using Api 20 Strep. Eleven of the 22 *E. faecalis* strains were also identified as *E. faecalis* using Api 20 Strep.

All 100 strains isolated from the river water samples (Haruki, Sakai, and Asami rivers, and urban drainage) were analyzed using the same identification tests. In the Haruki River, the number of strains identified as *E. faecalis* was 6 times higher than that identified as *E. faecium*, thus showing a considerable difference. On the other hand, similar numbers of *E. faecium* and *E. faecalis* were identified in Sakai and Asami rivers and urban drainage. The smallest number of *E. faecium* and *E. faecalis* strains was identified in the Asami River. *E. faecium* and *E. faecalis* strains were identified from the water samples at all stations, and the proportions of these *Enterococcus* species varied among the stations. Finally, the number of *Enterococcus* strains identified as *E. faecium* and *E. faecalis* was 89 and 11, 10 and 63, 27 and 28, 20 and 28, and 9 and 8 in the water samples collected from the beach, Haruki River, Sakai River, urban drainage, and Asami River, respectively.

The genus *Enterococcus* includes opportunistic pathogens that normally inhabit human and animal gastrointestinal tracts and feces. *E. faecium* and *E. faecalis* have been consistently identified as the dominant *Enterococcus* spp. in the *Enterococcus* flora of human feces (approximately 90%) (12). Fecal pollution can be broadly classified into human and nonhuman pollution. The most serious threat to human health is thought to be from human rather than animal feces in water environments (3). Therefore, *E. faecium* and *E. faecalis* are very important species for evaluating fecal pollution in water environments. Identification of *E. faecium* and *E. faecalis* using PCR and Api 20 Strep showed that these two *Enterococcus* species were found in water samples from all stations. The total proportion of *E. faecium* and *E. faecalis* in each water sample was >50%, except in the water sample from Asami River. This indicated that all sampling stations (the beach, Hariki and Sakai rivers, and urban drainage), with the exception of Asami River, might be polluted by fecal bacteria. *E. faecium* was the dominant species in samples from the beach. In contrast, *E. faecalis* was identified at much higher levels than *E. faecium* in the samples from the three rivers and urban drainage. This suggests that the tolerance to fluctuating environmental conditions, i.e., freshwater and seawater, may differ between these two *Enterococcus* species. Further studies are required to determine the environmental fate of these species.

### Genotypic analysis of *E. faecium* and *E. faecalis* using PFGE

All 155 *E. faecium* and 138 *E. faecalis* strains isolated from the water samples were analyzed by PFGE using *Sma*I restriction enzyme. Many of the *E. faecium* strains isolated from the beach were analyzed by PFGE, but these strains only produced 12 PFGE types (Table 3). Of the 12 PFGE types of *E. faecium* isolated from the beach (72 strains), the largest number had the Fm-Rb-3 type, *i.e.*, the dominant PFGE type from the beach. In contrast, *E. faecium* strains isolated from each river water sample produced a wider variety of PFGE types compared with those isolated from the beach. In Haruki and Asami rivers, although the number of *E. faecium* strains isolated from both rivers was relatively low, various PFGE types were observed. *E. faecium* strains from both rivers had individual genotypes.

*E. faecalis* strains isolated from the beach (11 strains), Haruki River (63 strains), Sakai River (28 strains), urban drainage (28 strains), and Asami River (8 strains) produced 9, 31, 25, 22, and 6 PFGE types, respectively (Table 3). Thus, 93 PFGE types were found for the *E. faecalis* strains. The number of *E. faecalis* strains isolated from all stations was lower than that of *E. faecium* strains. However, the number of PFGE types of *E. faecalis* was more than that of the PFGE types of *E. faecium*.

### Similarity analysis of genotypes using dendrogram

**Fig. 2:**
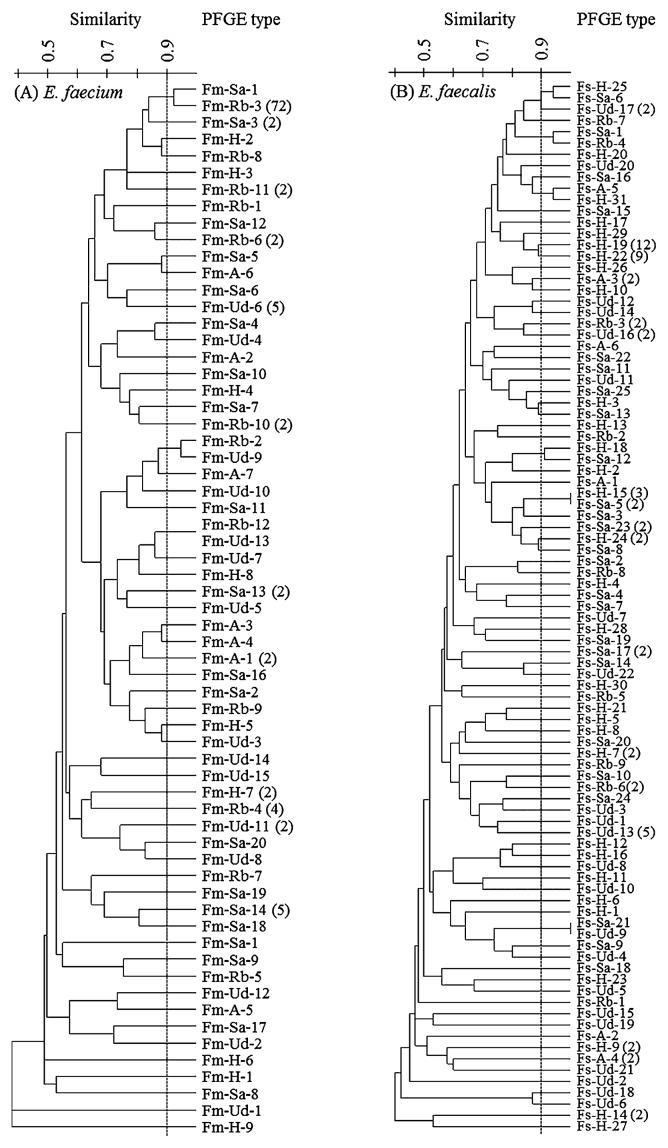
Dendrograms of all PFGE types of *E. faecium* (A), and *E. faecalis* (B). Rb: Recreational Beach, H: Haruki River, Sa: Sakai River, Ud: Urban drainage, A: Asami River. Numbers in parentheses are the strains that showed each band pattern. In the remaining PFGE types (no numbers in parentheses), only one strain showed these band patterns. Dashed line means the cut-off level of similarity in the dendrograms (0.9).

In this study, we analyzed the similarities of all *Sma*I-digested band patterns obtained from *Enterococcus* strains using dendrogram analysis. Dendrograms were constructed containing all the PFGE types of each *E. faecium* (Fig. 2A) and *E. faecalis* (Fig. 2B) strain. We determined the PFGE profiles at a 0.9 similarity level in the dendrogram containing *E. faecium* strains by classifying the PFGE types of Fm-Sa-1 obtained from Sakai River and those of Fm-Rb-3 (dominant type) obtained from the beach in the same cluster. The PFGE types of *E. faecium* that were classified into the same clusters at a 0.9 similarity level were closely associated with each other. The two PFGE types of the strains isolated from urban drainage were also classified in the same clusters as those from the beach at a 0.9 similarity level (Fm-Ud-9 and Fm-Rb-2, and Fm-Ud-3 and Fm-Rb-12). Thus, it was highly possible that fecal pollution of the beach was closely related with Sakai River and urban drainage. The PFGE types of the strains isolated from Haruki and Asami rivers were not classified into the same cluster as those from the beach.

There were a variety of PFGE types for *E. faecalis* strains, but these were not very similar. Only the PFGE type of Fs-Sa-1 obtained from Sakai River had more than 0.9 similarity with that of Fs-Rb-4 obtained from the beach. Meanwhile, some genotypes of *E. faecalis* strains isolated from different river water samples were classified into the same clusters (Fs-H-25, Fs-Sa-6 and Fs-Ud-17, Fs-A-5 and Fs-H-31, Fs-H-18 and Fs-Sa-12, Fs-H-15 and Fs-Sa-5, and Fs-Sa-21 and Fs-Ud-9). It was suggested that *E. faecalis* strains were derived from similar hosts in different rivers. Therefore, it seemed difficult to estimate the source of fecal pollution using the dendrogram of the genotypes obtained from *E. faecalis* strains in this coastal urban area.

Based on the results of the dendrogram analysis of *E. faecium* genotypes, the most likely sources of fecal pollution of the beach were Sakai River and urban drainage, although only one sampling was conducted for this demonstration of the MST approach. It was clear that this MST approach using PFGE can discriminate within the same species regardless of the differences among the *Enterococcus* spp. and the numbers of analyzed strains. A larger number of enterococci were detected from Haruki and Asami rivers than were detected from Sakai River and urban drainage, which were presumed to be the source of fecal pollution based on genotypic analysis using PFGE. Thus, rivers with the highest concentration of enterococci are not always the source of fecal pollution in coastal recreation areas.

An important consideration common to library-dependent methods is the response to temporal changes in the genotypes of fecal pollution indicators. Temporal change in genotypes is a factor that may complicate the determination and understanding of fecal pollution sources (10). A previous study reported that *E. coli* varied in PFGE banding patterns, which might be caused by mutation or recombination over the course of 8 weeks (15). It is important to clarify that temporal changes in the genotypes of indicators because of chemical and bacterial water quality and bacterial flora in rivers and coastal areas may be markedly changed by various factors such as rainfall, wind, and water current. In this study, to eliminate the problem of temporal changes in PFGE banding patterns of *Enterococcus* spp. as an indicator of fecal pollution, sampling was performed on a single day (July 21, 2009). As a result, it was shown that MST using PFGE could identify rivers that were fecal pollution sources of the beach. Bacterial water quality and *Enterococcus* flora in river water and seawater might change with the season, even in the same water environments, and therefore, multiple demonstrations of MST using PFGE will be essential in further studies. In addition, we will also try to identify more detailed sources of fecal pollution by the same MST approach within several stations such as municipal sewage, septic tanks, and livestock, which were selected as candidate sources in rivers that were considered as fecal pollution sources in the present study.

## Figures and Tables

**Table 1:** Major water parameters and bacterial counts in sample water

Sampling station	pH	Water temperature (°C)	Salinity (psu)	Turbidity (degree, kaolin)	Enterococci (CFU 100 mL^−1^)	Coliforms	E. coli

						(MPN 100 mL^−1^)	

					Mean±SD (*n*=3)	Mean	Mean
Recreational Beach	7.7	28.3	28.4	1.7	2.6×10^2^±10.8	63	0
Haruki River	7.6	28.7	0.2	8.9	5.2×10^2^±48.1	2.5×10^2^	10
Sakai River	7.4	28.6	0.1	12.2	93±15.3	3.7×10^2^	85
Urban drainage	7.3	28.6	0.1	7.8	67±45.1	3.3×10^2^	97
Asami River	7.7	28.1	0.2	18.4	3.8×10^2^±75.1	3.3×10^2^	62

**Table 2:** Number of *Enterococcus* strains identified as *E. faecium* and *E. faecalis* using PCR and Api 20 Strep

Sample	No. of isolated *Enterococcus* strains	No. of strains identified (strain)			
		*E. faecium*		*E. faecalis*	

		PCR	Api 20 Strep^a^	PCR	Api 20 Strep
Recreational beach	150	94	89	22	11
Haruki River	100	11	10	73	63
Sakai River	100	33	27	31	28
Urban drainage	100	23	20	30	28
Asami River	100	11	9	9	8
Total	550	172	155	165	138

a Api 20 Strep was carried out with regard to only *Enterococcus* strains identified *E. faecium* or *E. faecalis* by PCR.

**Table 3:** Number of PFGE types obtained from *E. feacium* and *E. faecalis* strains in all water samples

Sample	*E. faecium*			*E. faecalis*		
	
	No. of strains	No. of PFGE types	PFGE types	No. of strains	No. of PFGE types	PFGE types
Recreational Beach	89	12	Fm-Rb-1–Fm-Rb-12	11	9	Fs-Rb-1–Fs-Rb-9
Haruki River	10	9	Fm-H-1–Fm-H-9	63	31	Fs-H-1–Fs-H-31
Sakai River	27	20	Fm-Sa-1–Fm-Sa-20	28	25	Fs-Sa-1–Fs-Sa-25
Urban drainage	20	15	Fm-Ud-1–Fm-Ud-15	28	22	Fs-Ud-1–Fs-Ud-22
Asami River	9	7	Fm-A-1–Fm-A-7	8	6	Fs-A-1–Fs-A-6

## References

[1] Blanch AR, Belanche-Munoz L, Bonjoch X (2006). Integrated analysis of established and novel microbial and chemical methods for microbial source tracking. Appl Environ Microbiol.

[2] Chu G, Vollrath D, Davis RW (1986). Separation of large DNA molecules by contour-clamped homogeneous electric field. Science.

[3] Field KG, Samadpour M (2007). Fecal source tracking, the indicator paradigm, and managing water quality. Water Res.

[4] Figueras MJ, Borrego JJ, Pike EB, Robertson W, Ashbolt N, Bartram J, Rees G (2000). Chapter 8 Sanitary inspection and microbiological water Quality. Monitoring Bathing Waters—A Practical Guide to the Design and Implementation of Assessments and Monitoring Programmes.

[5] Furukawa T, Takahashi H, Yoshida T, Suzuki Y (2011). Genotypic analysis of enterococci isolated from fecal-polluted water from different sources by pulsed-field gel electrophoresis (PFGE) for application to microbial source tracking. Microbes Environ.

[6] Furukawa T, Yoshida T, Suzuki Y (2011). Application of PFGE to source tracking of fecal pollution in coastal recreation area: A case study in Aoshima Beach, Japan. J Appl Microbiol.

[7] Goering RV, Winters MA (1992). Rapid method for epidemiological evaluation of gram-positive cocci by field inversion gel electrophoresis. J Clin Microbiol.

[8] Gordon KV, Brownell M, Wang SY (2013). Relationship of human-associated microbial source tracking markers with Enterococci in Gulf of Mexico waters. Water Res.

[9] Griffith JF, Weisberg SB, McGee CD (2003). Evaluation of microbial source tracking methods using mixed fecal sources in aqueous test samples. J Water Health.

[10] Harwood VJ, Brownell M, Wang S (2009). Validation and field testing of library-independent microbial source tracking methods in the Gulf of Mexico. Water Res.

[11] Jackson CR, Fedorka-Cray PJ, Barrett JB (2004). Use of a genus- and species-specific multiplex PCR for identification of enterococci. J Clin Microbiol.

[12] Koujima I (1991). Distribution of enterococci as an indicator of water pollution. Jap J Water Treat Biol.

[13] Liu D, Wang C, Swiatlo EJ, Lawrence ML (2005). PCR amplification of species-specific putative transcriptional regulator gene reveals the identity of *Enterococcus faecalis*. Res Microbiol.

[14] Lleò MM, Bonato B, Benedetti D, Canepari P (2005). Survival of enterococcal species in aquatic environments. FEMS Microbiol Ecol.

[15] Lu L, Hume ME, Sternes KL, Pillai SD (2004). Genetic diversity of *Escherichia coli* isolates in irrigation water and associated sediments: implications for source tracking. Water Res.

[16] Manero A, Vilanova X, Cerda-Cuellar M, Blanch AR (2002). Characterization of sewage waters by biochemical fingerprinting of *Enterococci*. Water Res.

[17] Marti R, Gannon VPJ, Jokinen C (2013). Quantitative multi-year elucidation of fecal sources of waterborne pathogen contamination in the South Nation River basin using Bacteroidales microbial source tracking markers. Water Res.

[18] Martines-Urtaza J, Liebana E (2005). Investigation of clonal distribution and persistence of *Salmonella* Seftenberg in the marine environment and identification of potential source of contamination. FEMS Microbiol Ecol.

[19] Maslow JN, Slutsky AM, Arbeit RD, Persing DH, Smith TC, Tenover FC, White TJ (1993). Application of pulsed-field gel electrophoresis to molecular epidemiology. Diagnostic molecular microbiology, principles and applications.

[20] Matsushita Y, Takeuchi H (1986). Chapter 3 Determination of chemical parameters 3–6 Salinity. Encyclopedia of Measurement and Analysis on Global Environment, Vol. 3 Marine coastal environment.

[21] McQuaig S, Griffith J, Harwood VJ (2012). Association of fecal indicator bacteria with human viruses and microbial source tracking markers at coastal beaches impacted by nonpoint source pollution. Appl Environ Microbiol.

[22] Meays CL, Broersma K, Nordin R, Mazumder A (2004). Source tracking fecal bacteria in water: a critical review of current methods. J Environ Manage.

[23] Murugan K, Prabhakaran P, Al-Sohaibani S, Sekar K (2011). Identification of source of faecal pollution of Tirumanimuttar River, Tamilnadu, India using microbial source tracking. Environ Monit Assess.

[24] Parveen S, Murphree RL, Edmiston L, Kaspar CW, Portier KM, Tamplin ML (1997). Association of multiple-antibiotic resistance profiles with point and nonpoint sources of *Escherichia coli* in Apalchicola Bay. Appl Environ Microbiol.

[25] Robertson LJ, Forberg T, Hermansen L, Gjerde BK, Alvsvåg JO, Langeland N (2006). *Cryptosporidium parvum* infections in Bergen, Norway, during an extensive outbreak of waterborne Giardiasis in autumn and winter 2004. Appl Environ Microbiol.

[26] Santo Domingo JW, Bambic DG, Edge TA, Wuertz S (2007). Quo vadis source tracking? Towards a strategic framework for environmental monitoring of fecal pollution. Water Res.

[27] Sclichting C, Branger C, Fournier JM, Witte W, Boutonnier A, Wolz C, Goullet P, Döring G (1993). Typing of *Staphylococcus aureus* by pulsed-field gel electrophoresis, zymotyping, capsular typing, and phage typing: resolution of clonal relationships. J Clin Microbiol.

[28] Scott TM, Rose JB, Jenkins TM, Farrah SR, Lukasik J (2002). Microbial source tracking: current methodology and future directions. Appl Environ Microbiol.

[29] Stoeckel DM, Mathes MV, Hyer KE (2004). Comparison of seven protocols to identify fecal contamination sources using*Escherichia coli*. Environ Sci Technol.

[30] Tenover FC, Arbeit RD, Goering RV, Mickelsen PA, Murray BE, Persing DH, Swaminathan B (1995). Interpreting chromosomal DNA restriction patterns produced by pulsed-field gel electrophoresis: Criteria for bacterial strain typing. J Clin Microbiol.

[31] U.S Environmental Protection Agency (1986). Ambient Water Quality Criteria for Bacteria.

[32] US Environmental Protection Agency (2002). Method 1600: Enterococci in Water by Membrane Filtration Using membrane-Enterococcus Indoxyl-β-D-Glucoside Agar (mEI).

[33] US Environmental Protection Agency 2003 Bacterial Water Quality Standards for Recreational Waters (freshwater and marine waters) EPA-823-R-03-008US EPAWashington DC.

[34] US Environmental Protection Agency (2005). Microbial Source Tracking Guide Document.

